# The relationship between laboratory safety climate and hazardous incidences among laboratory staff selected health facilities in Kenya: preliminary results from an on-going national health care workers survey

**DOI:** 10.1186/2047-2994-4-S1-P98

**Published:** 2015-06-16

**Authors:** J Osoga, B Burmen

**Affiliations:** 1 HIV Implementation Science and Services, KEMRI-CGHR, Kisumu, Kenya

## Introduction

Hospitals’ safety climates have been correlated with incidents of exposure to blood and body fluids.

## Objectives

We examined the relationship between laboratory safety climates and hazardous incidents among laboratory personnel at selected health facilities in Kenya.

## Methods

A survey on history of hazardous incidents and safety mechanisms was conducted among laboratory personnel in Kenya. A hazard was defined as a fall, inhalation of harmful gas, ingestion of hazardous agents, subcutaneous chemical exposure, sharp injury or a hazardous spill. The laboratory safety climate was rated on a ten point scale. We used univariate analyses to describe the relationship between experiencing a hazard and the safety climate.

## Results

One hundred and thirteen laboratory personnel were interviewed; the median duration of service was 4.0 years (range 0.2 - 33 years), 62 (53%) had received Hepatitis B vaccination and 18 (16%) had been previously trained on biosafety. Health facilities were equipped with; sharps’ disposal facilities (90%), PPE (82%), waste disposal mechanisms (60%), containment of hazardous wastes (38%), fire safety equipment (38%), vaccination measures (28%), reporting mechanisms for exposures (22%), safety equipment (19%), protocols for occupational injuries documentation (17%) and safety audits (10%). Eighty-one (77%) laboratory personnel had experienced a hazard. Having been vaccinated against Hepatitis B, trained on biosafety, availed to safety mechanisms and work duration were not associated with history of a hazard.

## Conclusion

Although the relationship between hazardous incidents and safety mechanisms is not of statistical significance, the absence of appropriate laboratory safety mechanisms it is still of public health concern. Equipping labs with necessary safety infrastructure as well as assessing personal factors related to injuries may decrease the incidences of laboratory hazards.

## Disclosure of interest

None declared.

